# Supplementary material for: Patient reported postoperative pain with a smartphone application: A proof of concept
Source: PLoS One. 2020 May 8;15(5):e0232082. doi: 10.1371/journal.pone.0232082 (PMC7209286; doi:10.1371/journal.pone.0232082)
Supplement: S1 File — (DOCX) [file pone.0232082.s001.docx]

**Vragenlijst gebruikers OLVG PIJN app**

Geachte heer, mevrouw,

U bent gevraagd de OLVG PIJN app te beoordelen. De OLVG PIJN app is een smartphone applicatie waarmee patiënten in het ziekenhuis zelf pijn kunnen meten na een operatie. Om deze app te verbeteren is uw mening en commentaar nodig. Daarom vragen wij u deze korte vragenlijst in te vullen. Dit zal ongeveer 10 minuten duren.

Subject no. (in te vullen door onderzoeker):

Android □ of Iphone □ (in te vullen door onderzoeker):

| Deze vraag gaat over uw tevredenheid. Geef de mate van uw tevredenheid aan. |
| --- |
| **Hoe vond u het om met deze app de mate van pijn aan ons door te kunnen geven?**  Heel erg fijn □ Prettig □ Oké □ Niet prettig □ Zeer onprettig □ |

| Deze vraag gaat over de pijn na uw operatie. Geef de mate van pijn na uw operatie aan. |
| --- |
| **Hoeveel last heeft u gehad van pijn na deze operatie?**  (Bijna) niet □ Weinig □ Goed uit te houden □ Flinke pijn □ Hevige pijn □ |

| Deze stellingen gaan over het gebruiksgemak van de app. Geef aan in welke mate u het eens bent met deze stellingen. |
| --- |
| **Ik vind de app makkelijk te gebruiken.**  Helemaal mee oneens □ Oneens □ Geen mening □ eens □ Helemaal mee eens □  **Met de app kan ik mijn pijn goed doorgeven.**  Helemaal mee oneens □ Oneens □ Geen mening □ eens □ Helemaal mee eens □  **Ik vind het handig dat de app mij eraan herinnert om door te geven hoeveel pijn ik heb.**  Helemaal mee oneens □ Oneens □ Geen mening □ eens □ Helemaal mee eens □ |

| De volgende vragen gaan over hoe aantrekkelijk u de app vindt. U geeft een waardering op een schaal van 1 tot 5 waarbij 1 erg onaantrekkelijk is en 5 erg aantrekkelijk is. |
| --- |
| **Hoe vindt u de app eruit zien?**  Erg onaantrekkelijk : 1 □ 2 □ 3 □ 4 □ 5 □ : Erg aantrekkelijk  **Wat vindt u van de gebruikte kleuren in de app?**  Erg onaantrekkelijk : 1 □ 2 □ 3 □ 4 □ 5 □ : Erg aantrekkelijk  **Wat vindt u van het gebruikte lettertype in de app?**  Erg onaantrekkelijk : 1 □ 2 □ 3 □ 4 □ 5 □ : Erg aantrekkelijk  **Wat vindt u van de indeling van de app?**  Erg onaantrekkelijk : 1 □ 2 □ 3 □ 4 □ 5 □ : Erg aantrekkelijk |
| De volgende vragen gaan over de inhoud van de app. In welke mate vindt u deze bruikbaar. U geeft een waardering op een schaal van 1 tot 5 waarbij 1 geheel niet bruikbaar is en 5 heel goed bruikbaar is. |
| **Hoe vindt u het invullen van uw pijnscore in de app?**  Geheel niet bruikbaar : 1 □ 2 □ 3 □ 4 □ 5 □ : Heel goed bruikbaar  **Zou u een verpleegkundige met de app op willen roepen?**  Geheel niet bruikbaar : 1 □ 2 □ 3 □ 4 □ 5 □ : Heel goed bruikbaar  **Vindt u de instructie film duidelijk?**  Geheel niet bruikbaar : 1 □ 2 □ 3 □ 4 □ 5 □ : Heel goed bruikbaar  **Wat vindt u van de grafiek die de app laat zien?**  Geheel niet bruikbaar : 1 □ 2 □ 3 □ 4 □ 5 □ : Heel goed bruikbaar |

| Tot slot nog enkele open vragen. |
| --- |
| Zou u iets willen toevoegen aan de app om deze nog handiger te maken?  Zijn er onderdelen in de app die u overbodig vindt? |

Hartelijk dank voor het beantwoorden van deze vragen. Met uw suggesties en opmerkingen kunnen wij de app verbeteren.
