# Supplementary material for: Patient reported postoperative pain with a smartphone application: A proof of concept
Source: PLoS One. 2020 May 8;15(5):e0232082. doi: 10.1371/journal.pone.0232082 (PMC7209286; doi:10.1371/journal.pone.0232082)
Supplement: S2 File — (DOCX) [file pone.0232082.s002.docx]

**Patient questionnaire OLVG Hospital PAIN application (translated from Dutch)**

Dear sir/madam,

You are being asked to evaluate the OLVG Hospital pain smartphone application. This application provides patients a tool to self record their pain after surgery. To improve this application your opinion and comments are necessary. That’s why we ask you to fill in this questionnaire it will take you approximately 10 minutes

Subject no.:

Android □ or IPhone □:

| This question is about your satisfaction. Please rate your satisfaction. |
| --- |
| **Q1 How did you like to report your pain with this app?**  Very pleasant □ Pleasant □ Ok □ Not pleasant □ Very unpleasant □ |

| This question is about the pain you experienced after surgery. Please rate your overall postoperative pain. |
| --- |
| **Q2 How much pain have you had after surgery?**  No pain at all □ Mild pain □ Bearable pain □ Not bearable □ Severe pain □ |

| These statements are about the ease of use. State to what extent you agree with these statements. |
| --- |
| **Q3 I think the app is easy to use.**  Totally disagree □ Disagree □ Neutral □ Agree □ Totally agree □  **Q4 With the app I can record my pain well.**  Totally disagree □ Disagree □ Neutral □ Agree □ Totally agree □  **Q5 I find it useful that the app notifies me to record my pain.**  Totally disagree □ Disagree □ Ok □ Agree □ Totally agree □ |

| The following questions are about the design of the app. You have to rate the design of the app on a scale from 1 to 5. |
| --- |
| **Q6 How attractive did you find the look of the app overall?**  Very unattractive : 1 □ 2 □ 3 (ok) □ 4 □ 5 □ : Very attractive  **Q7 How attractive did you find the use of color in the app?**  Very unattractive : 1 □ 2 □ 3 (ok) □ 4 □ 5 □ : Very attractive  **Q8 How attractive did you find the fonts used in the app?**  Very unattractive : 1 □ 2 □ 3 (ok) □ 4 □ 5 □ : Very attractive  **Q9 How attractive did you find the layout of the app?**  Very unattractive : 1 □ 2 □ 3 (ok) □ 4 □ 5 □ : Very attractive |
| The following questions are about the content of the app and if it suits the purpose of the app. You have to rate the content on a scale from 1 to 5. |
| **Q10 How sufficient did you feel the content of the app was in enabling you to record your pain?**  Not useful at all : 1 □ 2 □ 3 (ok) □ 4 □ 5 □ : Very useful  **Q11 Would you like to call for a nurse with the app?**  Not useful at all : 1 □ 2 □ 3 (ok) □ 4 □ 5 □ : Very useful  **Q12 How do you like the in-application the pain intensity chart?**  Not useful at all : 1 □ 2 □ 3 (ok) □ 4 □ 5 □ : Very useful |

| Some final questions. |
| --- |
| Would you like to add something in the app to make it more useful?  Are there items in the app which you find unnecessary? |

Thank you very much for answering these questions. With your comments we can improve the app.
