# Supplementary material for: Patient reported postoperative pain with a smartphone application: A proof of concept
Source: PLoS One. 2020 May 8;15(5):e0232082. doi: 10.1371/journal.pone.0232082 (PMC7209286; doi:10.1371/journal.pone.0232082)
Supplement: S3 File — (DOCX) [file pone.0232082.s003.docx]

Feedback specialist: Johan Haumann

Datum: 16-10-2017

iOS V1.0.0 (11)

1: eerst app laten gebruiken en kijken wat er gebeurd zonder iets te vertellen.

2: Laat mensen eerst een paar keer de vragen langs gaan en kijken naar de grafiek.

**Grafisch/interface**

-Hoe ziet de app eruit?

-Wat vind je van de kleuren?

-Wat vind je van het lettertype?

-Wat vind je van de lay-out?

-Oogt het professioneel?

-Wordt de gegeven informatie duidelijk gepresenteerd?

-Vind je het design passen bij de functie van de app?

-Verder commentaar over het design en uiterlijk?

Is allemaal prima

**Navigatie**

Is navigeren makkelijk (is alles goed te begrijpen en duidelijk voor de gebruiker?).

Gaat navigeren makkelijk? (is de app snel, werkt het goed)

Prima

**Pijn vragen**

-Zijn de vragen duidelijk en te begrijpen?

Ja , duidelijk en goed gericht op POP

Wat vind je van de pijn score vraag? Is de slider duidelijk/ goed te begrijpen?

Is duidelijk

-Geven ze u genoeg informatie als specialist? Wat zou je nog meer willen weten of wat zou je qua anders willen zien qua vragen?

Het zou handig zijn als de interventie die je doet ook in de grafiek staat. Bv. Als je extra medicatie geeft omdat de pijn door het normale pijn schema heen komt. Als je deze extra medicatie aangeeft als een event, dan kun je zien wat voor effect je behandeling heeft.

Wat ook handig is, is als patiënten kunnen aangeven dat ze hun medicatie juist niet hebben ingenomen. Zodat je ook hiervan ziet wat voor effect het geeft.

-Wat vind je van de feedback die je krijgt van de app, dus wat je van de app terug krijgt (bevestigingen etc)? Is de informatie voorziening door de app voldoende?

**Historie grafiek**

Wat vind je van de grafiek?

Prima

Word je door de grafiek van genoeg informatie voorzien over wat je in het verleden in hebt gevuld?

ja, behalve dat ik ook graag de interventies er in terug zou willen zien

Pijn van de gemiddelde patiënt in de grafiek?

Tijdens de opname lijkt het me niet van toegevoegde waarde voor de patiënt. Maar voor de operatie is het misschien wel handig om de gemiddelde grafiek aan patiënten te tonen. Je kan dan ook voorlichting geven hoelang ze ongeveer klachten zullen hebben.

**Conclusie**

-Zijn er zaken die je aan de app zou toevoegen om jouw pijn registratie als patiënt nuttiger te maken?

-Zijn er zaken die je aan de app zou toevoegen om de pijn registratie als specialist nuttiger te maken?

Last van bijwerkinen? Ja of nee en dan misschien een tekstbox met welke bijwerkingen. Of dat je de 7 standaard bijwerkingen van opiaten zou kunnen aanvinken, mocht je die als patiënt hebben.

-Zijn er onderdelen in de app die overbodig zijn?

-Zijn er zaken die je specifiek goed vind?

Het is een simpele en zeer handige app. Ik verbaas me er over dat het nog niet eerder is bedacht en al is uitgewerkt. Het lijkt me namelijk heel handig

-Zijn er zaken die je specifiek niet goed vind?

-Verder zaken die je opvielen of opmerkingen?

Wat heel handig zou zijn is als er ook een enigszins aangepaste App kan worden gemaakt voor chronische Pijn patiënten. Wat je dan zou kunnen is patiënten die app geven. Vervolgens krijgen deze patiënten dan een interventie, zoals bv een blok. En je kunt dan vragen of ze het de dagen erna 3 keer per dag kunnen invullen. Op deze manier zie je dan het effect van de interventie. Op het moment dat ze stabiel zijn kunnen ze het dan bv 1 keer per week invullen.
