# Supplementary material for: Patient reported postoperative pain with a smartphone application: A proof of concept
Source: PLoS One. 2020 May 8;15(5):e0232082. doi: 10.1371/journal.pone.0232082 (PMC7209286; doi:10.1371/journal.pone.0232082)
Supplement: S4 File — (DOCX) [file pone.0232082.s004.docx]

**Appendix II: Semi-structured interview Healthcare Professional**

Profession:

Date:

Operating system:

1: Let the interviewee use the application without explanation and observe if there are difficulties in the first use of the application

2: Let the interviewee review the pain questions and the in-app pain chart.

**Interface**

- What do you think of the lay-out and interface of the application?

- What do you think of the used colors?

- What do you think of the used font-type?

- Does the application have a professional look and feel?

- Is the in-app information about the use of the application clearly presented?

- Do you think the design suits the purpose of the application?

- Do you have any further comments on the design and interface?

**Navigating through the app**

-Is navigating through the application easy?

-Button response time?

-No errors or malfunctioning?

**In-app pain questions**

-Are the questions clear and unambiguous?

- What do you think of the pain-intensity slider? (Clear ‘end of scale’ ankers?)

- Do you expect the answers being given by patients provide you enough information as a health care professional?

-What other questions would you recommend?

- What do you think of the in-app feedback being given tot he patients?

**In-app pain chart**

-What do you think of the in-app pain chart?

-Is clear and comprehensive?

-Does the chart provide enough and detailed information?

**Conclusion**

- Are there any items that you would like to add to make pain recording with the app more useful for the patients?

- Are there any items that you would like to add to make pain recording with the app more useful for healthcare professionals?

- Are there items unnecessary?

- Are there items specifically okay?

- Are there items specifically not-okay?

- Do you have any final remarks?
