# Supplementary material for: Patient reported postoperative pain with a smartphone application: A proof of concept
Source: PLoS One. 2020 May 8;15(5):e0232082. doi: 10.1371/journal.pone.0232082 (PMC7209286; doi:10.1371/journal.pone.0232082)
Supplement: Attachment — Submitted filename: Rebuttal letter_6_10_2019.docx [file pone.0232082.s007.docx]

Rebuttal letter: PONE-D-17106

Title: Patient reported postoperative pain with a smartphone application: a proof of concept

Date: October 6^th^ 2019

Dear Editorial Board, Dear Erik Loeffen,

On behalf of all authors who contributed to this manuscript I thank you for the opportunity to submit a revised version. We thank the editor and both reviewers for their valuable remarks and comments. In this rebuttal letter we will address all comments separately. We would also like to mention that the tables and reference list are only correct in the clean version copy, as we made changes to it which could not adequately be processed in the track changes version.

**Editorial comments:**

*To enhance the reproducibility of your results, we recommend that if applicable you deposit your laboratory protocols in protocols.io.*

A summary of the study protocol is published in the Dutch trail register www.trialregister.nl. The Nederlands Trial Register (NTR) is a publicly accessible and freely searchable register in which current prospective studies run in the Netherlands or that are carried out by Dutch researchers. The study registration number is: NL6565. We added the following sentences to the manuscript: “The study is registered with a summary of the study protocol in de Dutch Trial Register (Nederlands Trial Register) with number NL6565”, accompanied with the NTR website URL in the reference list. We think this provides enough information for researchers who are interested in the protocol and the study method.

*When submitting your revision, we need you to address these additional requirements (point 1)*.

We corrected and renamed the files for figures, tables and supplemental materials.

*Please include additional information regarding the survey or questionnaire used in the study and ensure that you have provided sufficient details that others could replicate the analyses. For instance, if you developed a questionnaire as part of this study and it is not under a copyright more restrictive than CC-BY, please include copies, in both the original language and English, as Supporting Information (point 2).*

The survey is not under a copyright. We derived questions and answer options from the study of Reynoldson et al 2014 and translated them into Dutch ^1^. This has already been mentioned in the original manuscript (page 8). The survey is uploaded as a Supporting Information file in Dutch (original language) and English.

*Please note that PLOS ONE has specific guidelines on software sharing for manuscripts whose main purpose is the description of a new software or software package. In this case, new software must conform to the Open Source Definition and be deposited in an open software archive (point 3).*

We understand the importance of sharing software. The objective of our study is to collect patient and stakeholder recommendations to improve the prototype pain application and to evaluate if patient self-reporting with an application is a workable method for postoperative pain management. We have no intentions to study and specify the technical background of the application. The application is tailored for OLVG hospital. The ‘source code’ of the application cannot be made publicly because of security and the risk of abuse by third parties.

*Thank you for stating the following in the Competing Interests section: "I have read the journal's policy and the authors of this manuscript have the following competing interests: Rover A.A.M. van Mierlo is employee at Logicapps Inc. the company that built the application commissioned by OLVG. OLVG and Logicapps have no commercial interest in the application. The application is available free of charge for patients. "*

*Please confirm that this does not alter your adherence to all PLOS ONE policies on sharing data and materials, by including the following statement: "This does not alter our adherence to PLOS ONE policies on sharing data and materials.” (as detailed online in our guide for authors http://journals.plos.org/plosone/s/competing-interests). If there are restrictions on sharing of data and/or materials, please state these. Please note that we cannot proceed with consideration of your article until this information has been declared.*

*Please include your updated Competing Interests statement in your cover letter; we will change the online submission form on your behalf (point 4).*

We confirm the following statement ‘This does not alter our adherence to PLOS ONE policies on sharing data and materials’ and added it to the new cover letter. We are very grateful if you change the online submission form on our behalf as you suggested. Furthermore, it was never our intention not to be transparent about the CoI. Removing the author seemed the best option to make the submission possible and was done at the insistence of Van Mierlo himself. We kept Van Mierlo listed as author without omitting the clarity of his competing interests.

*In your Data Availability statement, you have not specified where the minimal data set underlying the results described in your manuscript can be found. PLOS defines a study's minimal data set as the underlying data used to reach the conclusions drawn in the manuscript and any additional data required to replicate the reported study findings in their entirety. All PLOS journals require that the minimal data set be made fully available (point 5).*

We uploaded the study’s minimal underlying data set as a Supporting Information file.

**Reviewer comments:**

*Reviewer #1: In the future including the question, do you need additional pain medication? Could close the loop in assessment and treatments.*

We agree with reviewer #1 that it is important to close the loop in pain assessments and treatments. In the version of the application that we are currently working on we added a similar question: ‘Should something be done about your pain?’ The formulation of this question is based on semantic arguments. Using the terms ‘additional pain medication’ could give the expectation to patients that pain medication always is an option and available. In reality, there are also other interventions such as, psychological support, posture and exercise advice, and applying heat and comfort. Closing the loop between patients and nurses does require in a technical solution so that the patient can contact or warn the nurse with the application this does require a technical solution which is not yet provided.

*Reviewer #1: As the authors note…record their pain by answering the following question ‘are you in pain; at rest or during movement? Furthermore, they could define the severity of pain on a scale from 0 (no pain) to 10 (worst imaginable pain)…There is typically a large difference between pain at rest and pain with activities. Depending upon the surgery, a VAS or NRS of 4 or more points…´It was not always clear whether they should rate their pain at rest or during movement…´The same may apply to the nurses’ assessments.*

To reviewer #1 this appears to be a limitation of the study but we see it is as a result from the study which we possibly have not clarified enough. This study was performed to collect patient and stakeholder comments to improve the application. One of the results is that patients are confused about whether they rate their pain in rest or during movement. One of the improvements of the new 2.0 version of the app is that it starts with the question ‘are you in pain?’ This is mentioned on page 19 of the manuscript.

*Reviewer #1: ‘’the patient’s valuation possibly leads to analgesic over-administration and may even contribute to opioid addiction which is a major problem in health care.’’ We have not yet established short term inpatient opioid use causing addiction. There are some associations but this is not yet established.*

We agree with reviewer #1 on this point and have revised this section of the manuscript.

*Reviewer#2: The introduction needs to clearly identify the weaknesses of common post-operative pain measures and measurement methods that are likely to be specifically addressed by the app.*

We have revised the introduction on several points.

*Reviewer#2: What reasons for not participating were given by the 31 patients who declined to enrol?*

According to Dutch legislation (Medical Research act). Patients can refuse to participate in medical research without giving reasons. Furthermore, if a patient refuses to participate, it is not allowed to document stated reasons and publish these. We do very much see the relevance for this manuscript in elucidating any potential (selection) bias but we regret we cannot and may not share the relevant data if we could.

*Reviewer#2: The methods describe that patients were not permitted to use their cell phones until their self-reported ratings were 4 or lower on a 0-10 pain scale.*

This is not correct, as already stated in the manuscript.’After surgery patients were discharged from the postoperative anesthesia care unit (PACU) under the condition that their pain was ‘acceptable’ with a numerical rating scale (NRS) lower or equal to 4. Back on the nursing ward, patients could start using the application unrestricted to self-report on their postoperative pain’.

Reviewer#2: *The results need to provide descriptive statistics-including minimum and maximum – for the number of ratings provided per patient before questionnaire completion. Some patients did not stay for an entire day in the hospital so it is possible they had very little experience using the app before completing the survey about the app.*

We agree with reviewer#2 that this provides a better insight into how long the patient has actually used the application. Unfortunately we have not asked patients to provide the questionnaire with data and time. The patient questionnaires were collected by one of the researchers on the day of discharge.

*Reviewer#2: Patients’ and nurses’ pain ratings were provided asynchronously so it does not make sense to test their agreement. In addition, why did the researchers choose to use nurses’ documented pain ratings as comparators to patients’ app ratings after they described how nurses alter patients’ self-reported pain ratings in the introduction?*

The statistical analysis for comparing the differences in patient and nurse pain recordings is done with non-parametric testing using Mann Whitney U test as stated in the manuscript. As reviewer#2 already concluded the pain ratings were provided asynchronously so we did not test for agreement. The Argument for using nurse pain recordings to compare with is that there is no other alternative; nurse pain recordings are still the gold standard for post-operative pain assessment

*Reviewer#2: regardless, nurse and patient agreement would be best compared by Bland Altman analysis with two groups of patients – one with the app and one with investigator recorded verbal pain ratings.*

We are not sure if we understand this comment. Bland-Altman analysis is the quantification of the agreement between two quantitative methods by studying the mean difference and constructing limits of agreement under the condition that both methods are designed to measure the same parameter and that measurements are taken on the same time point ^2^. Separate patient groups as suggested by reviewer#2 is in our opinion not possible.

*Reviewer#2: The app uses an 11-level faces pain scale. The manuscript provides no reference for the reliability and validity of this faces pain scale.*

The facial expression is added to the numerical rating scale to clarify the meaning of the numbers and the direction of the scale from 0 (no pain at all) to 10 (worst pain imaginable). The two scales are thus provided jointly. As we often see when we only use numerical rating scale many patients use the scale in reverse to rate their pain, as if to rate achievements and experiences with a number on a scale from 0 to 10, with 10 as the best positive result. We have added the following to the manuscript ‘The NRS was also equipped with an 11-face pain scale to clarify- and indicate the direction of the NRS. The use of an 11-face pain scale seems appropriate for measuring acute postoperative pain in adult patients as was shown in a study amongst orthopaedic surgical patients’ Provided with a suitable reference.

*Reviewer#2: The methods provide little detail on the procedure for interviewing the healthcare professionals. For example, were they interviewed individually or in groups? It appears they were asked to examine the app at the start of the interview. Was this examination meant to be comparable to patients’ experiences actually using the app?*

The stakeholders were interviewed individually. Indeed, they were asked to examine the application before the start of the interview. This was not meant to be comparable to patients’ experiences who could actually use the application for the time being admitted to hospital. We revised the methods section of the manuscript to provide more clarity.

*Reviewer#2: Rationales for the result focus on the times of pain ratings and division of the results by patient were not provided.* We agree with reviewer#2 that the manuscript provides no clarity about the rationales for the secondary objectives. The secondary objective was to investigate if patient self-recording compared to nurse-led assessment is a suitable method for postoperative pain management. We have adjusted the abstract, introduction and materials and methods to explain our rationales better.

*Reviewer#2: Manuscript needs to clarify the terms “pain intensity slider” and “pain chart” early.*

We corrected ‘pain intensity slider’ into ‘Numerical Rating Scale’ throughout the manuscript to provide more clarity. The in-app pain chart was already explained in the methods section with an accompanying image. We have not adjusted this.

*Reviewer#2: Abstract needs to clarify how patients’ and nurses’ pain ratings were “comparable”.*

We adjusted the abstract with the following sentence: The results suggest that the overall median pain intensity scores from patients recorded with a smartphone application compared with pain scores recorded by nurses’ show no statistical significant difference and therefore could be used for postoperative pain management.

*Reviewer#2: The terms “benchmark” and “benchmarking” appear throughout the manuscript without clear meaning.*

We have corrected the terms “benchmark” and “benchmarking” throughout the manuscript with appropriate reformulation.

*Reviewer#2: The meanings of “back-office system of Logicapps,” “ease of customization,” “CE certificate,” and “validation questions” need to be provided.*

We corrected ‘back-office system’ into ‘data server’. We corrected validation questions into ‘in-app pain assessment questions’. We corrected “ease of customization” into “ease of adjustment”

We explained ‘CE certificate’ in the manuscript as Conformite European certificate (CE certificate) this is a certificate to indicate conformity with health, safety and environmental protection standards for products used and sold within the European Economic Area (EEA) such a certificate is not mandated during development and research of a smartphone application.

*Reviewer#2: Correct “a CE certificate for was not necessary…”*

We added Conformite European certificate (CE certificate) and corrected the sentence.

We thank reviewer#2 for the comments: *The purpose of the references provided after the sentences describing sample size are unclear.* In this reference the theory behind the methods used to estimate the sample size are explained

*Reviewer#2: Throughout the manuscript, pain ratings are categorized (e.g., “considerable”) without any reference supporting the categorizations* In the patient questionnaire we have used the following categorization; “no pain” , “little pain”, “bearable pain”, “considerable pain”, “severe pain”. This was meant as a verbal rating scale for patients to value their overall experienced pain during the post surgical admission. The original Dutch terms used in the questionnaire are derived from the studies of Breivik et al 2008 ^3^ and Jensen et al 2011 ^4^. Currently there is no validated verbal rating scale for Dutch language available. We have stated this in the outcomes section of the revised manuscript.

*Reviewer#2: Are “bearable” and “acceptable” meant to be the same category of pain ratings?*

We exchanged ‘acceptable’ throughout the manuscript in ‘bearable’ in accordance with the request in the application and patient questionnaire.

*Reviewer#2: Are the “stakeholders” and “experts” also the “healthcare professionals”? Manuscript states “patients and stakeholders” and “patients and experts.” Participants, who were patient advisors, should not be described as “healthcare professionals” unless they were also licensed healthcare professionals.*

We corrected ‘healthcare professionals’ throughout the manuscript into ‘stakeholders’ and explained the composition of this group in the abstract and methods section.

*Reviewer#2: correct “from the patients and experts,” “chart does not,” “patients (green) record”.*

We corrected these sentences in the manuscript.

*Reviewer#2: The figures show the app asked if patients wanted additional medication, but they did show the app asking if the patients wanted nursing assistance as was stated in the manuscript.*

We are not sure if we understand this comment all screens of the application are uploaded as a figure. Possibly due to the Dutch language used in de figures there might be some misinterpretation, for the revised manuscript we translated all figures into English.

*Reviewer#2: Need to adhere to a guideline for reporting qualitative methods such as O’Brien et al. (2014) in addition to the STROBE guideline.*

We agree with reviewer#2 that the suggested reference provides a useful checklist for reporting. We used it as a final check before submitting the revised version of the manuscript, Furthermore, we added the following to the manuscript ‘the study results are reported according to the Strengthening the reporting of observational studies in epidemiology (STROBE) guideline for observational studies and Standards for reporting qualitative research (SRQR)’. O’Brien et al 2014 ^5^ has been added to the manuscript reference list.

*Reviewer#2: Manuscript refers to the reliability of the app, but the reliability of the app was not examined in the manuscript.*

We agree with reviewer#2, we did not test for reliability. We have changed the terms reliable and reliability into ‘comparable’ in several sections of the manuscript

*Reviewer#2: correct “Although the healthcare professionals suggested displaying analgesic medication, in the in-app pain intensity chart as well”*

We corrected this sentence into: ‘Yet, the stakeholders suggested that it would be useful displaying analgesic medication in the application pain chart to provide patients more information about their treatment’.

*Reviewer#2: All the figures need to be in English.*

We translated all figures to English where this was not sufficiently done so.

**References**

1. Reynoldson C, Stones C, Allsop M, et al. Assessing the quality and usability of smartphone apps for pain self-management. Pain medicine (Malden, Mass). 2014;15(6):898-909.
2. Bland JM, Altman DG, Measuring agreement in method comparison studies, Statistical Methods in Medical Research 1999, 8(2), 135–160. <https://doi.org/10.1177/096228029900800204>
3. Breivik H, Borchgrevink PC, Allen SM, et al. Assessment of Pain, British Journal of Anaesthesia 2008; 101(1);17-24
4. Jensen Hjermstad M, Fayers PM, Haugen DF, et al. Studies comparing numeric rating scales, verbal rating scales and visual analogue scale for assessment of pain intensity in adults: a systematic literature review. Journal of Pain and Symptom Management 2011;41(6):1073-93
5. O'Brien BC1, Harris IB, Beckman TJ, Reed DA, Cook DA, Standards for reporting qualitative research: a synthesis of recommendations, Acad Med. 2014 Sep;89(9):1245-51. doi: 10.1097/ACM.0000000000000388.

Sincerely yours, also on behalf of my co-authors

Bram Thiel, MSc

OLVG Hospital

Department of Anesthesiology

PO Box 95500; 1090 HM Amsterdam, the Netherlands

E: b.thiel@olvg.nl;

T: +31614584978
