# Supplementary material for: Patient reported postoperative pain with a smartphone application: A proof of concept
Source: PLoS One. 2020 May 8;15(5):e0232082. doi: 10.1371/journal.pone.0232082 (PMC7209286; doi:10.1371/journal.pone.0232082)
Supplement: Attachment — Submitted filename: Rebuttal letter_31_1_2020.docx [file pone.0232082.s008.docx]

Rebuttal letter: PONE-D-19-17106R1

Title: Patient reported postoperative pain with a smartphone application: a proof of concept

Date: January 31^st^ 2020

Dear Editorial Board, Dear Peter ten Klooster,

On behalf of all authors who contributed to this manuscript I thank you for the opportunity to submit a revised version. We are grateful to the editor and the reviewer for their valuable comments and suggestions for improvement. Below we address each comment separately.

**Editorial comments:**

*Please pay particular attention to and thoroughly check the accuracy of all statistics throughout the entire manuscript. The reviewer already pointed out several errors or inconsistencies between the abstract and the manuscript body. I also noted a potential error with the numbers in the abstract. For instance, in the abstract you described that “…Two patients (1%) were …”, whereas this percentage should probably be 4% considering the sample of 50 patients.*

The editor and the reviewer identified several inconsistencies in the descriptive statistics. We performed a thorough check of all descriptives in the abstract and the body of the manuscript. We found inconsistencies in the abstract, the body of the manuscript and table 2 and corrected these. From the abstract we have removed the following ‘Experienced pain after surgery was scored by patients as ‘no to mild’: 8 (16%), ‘bearable’: 25 (50%) and ‘considerable’ or ‘severe’: 24 (28%) (track changes lines 36-37).

In the body of the manuscript we corrected the following in line with table 2 ‘The overall experienced postoperative pain was valued as no pain by 3 patients (6%), or little pain in 5 patients (10%), 25 patients (50%) valued their pain as bearable and 13 (26%) valued their pain as considerable. One patient (2%) experienced severe pain’ (track changes lines 239-242).

In table 2 we have corrected the cell in row 12, column 3 from: 24 (48%) in 23 (46%). Thereby we also corrected this sentence from the results section: ‘The in-app pain intensity chart was valued as (very) useful by 41 patients (82%) in ‘The in-app pain intensity chart was valued as (very) useful by 40 patients (80%)’ (track changes lines 247-248).

*To enhance the reproducibility of your results, we recommend that if applicable you deposit your laboratory protocols in protocols.io*

As already mentioned in the previous rebuttal letter a summary of the study protocol is published in the Dutch trial register (www.trialregister.nl). The Dutch trial register is a publicly accessible and freely searchable register for prospective studies run in the Netherlands or that are carried out by Dutch researchers. The study registration number is: NL6565 (track changes lines 91-93).

**Reviewer comments:**

1.*The revisions to the manuscript have added the label of NRS or Numerical Rating Scale to describe the app pain scale used. This is just not accurate. The Numerical Rating Scale is not just using the numbers 0-10 to describe pain. It should include these numbers along a line, with the whole line visible at once. Often there are anchor words at either end. The app, as shown in Figure 1, does not do this. I assume a line with number is what is used by the nurse. If instead the nurse used some other form of the numbers or it was purely verbal, it would be good to add this to the manuscript. Your Kim et al 2012 citation from the Journal of Palliative Care clearly shows a line with numbers scale where you can see the whole line at once. Your first citation, for the NRS, Breivik et al. 2008 from BJA also shows a line with number for the NRS.*

*Please refrain from referring to the app scale as the NRS. Much work has been done to validate the proper NRS as a good pain scale. Is there any scientific evidence of this wheel scale (where you can only see a few numbers at once and the faces seem to be the focus) being consistent and valid as a pain scale? What was the starting position of the wheel? Was it 0, 5, or 10? This needs to be well explained or cited within the paper.*

We agree with the reviewer that referring to the pain app scale as numerical rating scale (NRS) is not appropriate. Indeed Breivik et al. 2008 shows a line with numbers for the NRS but they also mention in their paper ‘An NRS with numbers from 0 to 10 (‘no pain’ to ‘worst pain imaginable’) is more practical than a VAS, easier to understand for most people, and does not need clear vision, dexterity, paper, and pen. One can even determine the intensity of pain accurately using telephone interview, a computerized telephone interview, and recording of NRS data by the patient directly into the database of a computer via the telephone keyboard’ this is why we chose to refer to the pain app scale as NRS. In the manuscript we now use the neutral term *‘pain app scale’* and have corrected this throughout the manuscript.

The nurses in OLVG hospital routinely assess postoperative pain verbally by asking patients to indicate the severity of their pain with a number from 0 to 10, which is common practice in most Dutch hospitals. This is now mentioned in the manuscript : ‘Moreover, postoperative pain intensity was regularly verbally assessed on a scale from 0 (no pain) to 10 (worst imaginable pain) by ward nurses at least once every eight hours and thereafter recorded in the patient’s electronic medical record (EMR). This method of pain assessment is common practice in most Dutch hospitals and commonly referred to as ‘numerical rating scale’ (track changes lines 127-132). The starting position of the wheel was default set at 5, this in now mentioned in the manuscript: ‘The default starting position of the wheel was set at 5 and the wheel was provided with text anchors at both ends of the scale referring to no pain (0) and worst imaginable pain (10)’ (track changes lines 145-147).

*As seen though in Table 3, but not really touched on in the discussion, there is a moderately important comment from the stakeholders that the 11 faces look too similar and that the frequently used 6 face scale should be used. Yes, exactly, this is most likely referring to the Faces Pain Scale - Revised. Now, I understand you are using a new 11-faces pain scale to match the 11 numerical values. Amazingly, the paper that you cite for this, Van Giang et. al 2015 in Pain Management Nursing does not have an image of the scale. The original 11-face FPS paper, cited in that paper as Kim and Buschmann, 2016 in International Journal of Nursing Studies has 11 faces that appear to have more detail and look different than the very simple faces you used. Where did your faces come from? Were they designed by you for this app? That is not implied by your text. It would be best to use existing scales in the app to rely on some existing scientific evidence. Additionally, there is lots of good and critical feedback in Table 3 that can be used to improve your app. Perhaps once that is done, you can do a better test with it.*

We agree with the comments from the reviewer on both the pain scale and faces scale used the smartphone application. Both the pain scale and 11-faces scale were designed for this application. The faces scale used in the application was designed to fit with the 11 point numerical scale and was based on the 6 faces scale recommended by the 2015 pain guideline from the Dutch college of General Practitioners. This is now mentioned in the manuscript with reference (track changes lines 149-151). We disagree that it is not useful to test with both the pain scale and faces scale in this phase of developing an application. It is very important early in the design of eHealth tools to use end-user feedback to further improve the application one of the objectives of the study.

We agree that the used pain scale and 11-faces scale are not validated yet. We now mention this in the abstract and manuscript body ’Further research is needed to validate in-app pain scale and the 11-faces scale with the current gold standard VAS and NRS for pain’ (track changes lines 48-49) ‘More importantly, the pain intensity wheel has not yet been validated in relation to VAS or NRS, which are currently considered the gold standard for pain assessment (track changes lines 356-358) ‘Much work still needs to be done to thoroughly adjust and examine the psychometric features of the in-app pain intensity wheel and validate it against VAS and NRS’ (track changes lines 404-406).

2. *Leading from the above point, the second major problem is that the secondary objective is completely mixed up with the primary objective. That is, comparing the pain scores from this app with the nurse scores is not really meaningful, if you are now going to make large adjustments on the app - thus one of the two things you are comparing is disappearing (being greatly altered) in the future. If you are going to change the pain scale, which you should, then the comparison is not meaningful. I would suggest removing this secondary objective.*

We understand the reviewer on this point but we do not fully agree and we believe we have valid arguments to underpin this; the objective of this study is to collect data for improvement of the application and to see if patient self-recording of postoperative pain with a smartphone application could be used for postoperative pain management. We believe it is appropriate, even necessary, in this early stage of developing a smartphone application, to assess the agreement between patient-recorded and nurse-recorded pain scores, to better understand what is needed to improve and validate the used pain scale and faces scale.

*I also found the secondary objective results confusing, as you say that the patient and nurse scores are similar enough that one could substitute for the other (based on no significant difference in the medians) but then dive into the details of how certain ranges on the scales are significantly different.*

We agree with the reviewer that the results of the secondary objectives might confuse the reader. Therefore we have decided to remove the following paragraphs (including figure 5) from the manuscript to provide more clarity:

‘Patients record their pain more widely distributed throughout day and night (Figure 5). Nurses record pain mainly during their daily rounds, at 9:00, 12:00, and 18:00 and between 21:00 and 22:00. During the night, from 0:00 to 3:00, patients recorded pain 20 times and nurses recorded pain 4 times, respectively with median NRS 5 (range 1 to 9) and median NRS 6.5 (range 3 to 7) with a difference of 1.5 (p = 0.723). During the night, from 0:00 to 8:00, patients recorded their pain 54 times and nurses recorded pain 57 times, respectively with median NRS 5 (0 to 9) and median NRS 3 (0 to 8) with a difference of 2 (p = 0,03)’. Figure 5: Time distribution of pain score entries (track changes lines 303-310).

‘More importantly, the pain scores recorded by patients during the night were statistically significant higher compared with those recorded by nurses. Those patients with nightly high pain scores could potentially benefit from using the pain application, especially if we are able to establish a feedback loop from the app to the patients’ electronic medical record combined with an automated on-demand nurse warning system. However, in a recent study amongst 105 chronic pain patients, pilot testing a smartphone application with and without 2-way messaging between patient and healthcare provider. Patients in the 2-way massaging group felt that their healthcare providers were less responsive (29). Therefore it seems very important that the nurse is able to respond to the patients call when introducing 2-way messaging or a feedback loop in daily hospital care’ (track changes lines 342-351).

Minor issues:

3*. Please double check your numbers for the results. You have an error for the same number in the abstract and the manuscript body, assuming your table is correct. In the abstract*

*"‘severe’: 24 (28%)." should be "‘severe’: 14 (28%)."*

*In the body:*

*"and 30 (28%) valued their pain as considerable to severe" should be "and 14 (28%) valued their pain as considerable to severe"*

Thank you for pointing this out. We have corrected the descriptive statistics in the abstract (track changes lines 29-30) and the manuscript body (track changes lines 239-242), (track changes lines 247-248), according to the results from table 2.

4. *Please be clearer about where (and how many places) the study took place. You currently have:*

*"study was conducted in a Dutch general hospital situated at two locations in Amsterdam, the Netherlands"*

*Ok...so this is two locations of the same hospital? Or is this two areas of one hospital? In the discussion (limitations paragraph) you have:*

*"the study was performed in a single center setting"*

The study was conducted in OLVG Hospital which has two locations situated in different parts of Amsterdam. We adjusted the discussion section with the following sentences ‘This study is not without limitations. First, the study was performed on two locations of OLVG Hospital situated at different parts in Amsterdam which may not benefit the generalizability of the results’ (track changes lines 377-379).

5. Some typos/grammar problems in the manuscript:

*"...provided them instructions how to use the application..." should be:*

*"...provided them instructions on how to use the application..."*

This has been corrected (track changes line 108).

*"...A patient sample size of 50 patients was, based on sample size calculation for qualitative studies, was estimated..." should be:*

*"...A patient sample size of 50 patients, based on sample size calculation for qualitative studies, was estimated..."*

This has been corrected (track changes line 109).

*Figure 4: "adndroid" and "particoipate".*

This has been corrected, together with other adjustments of the figure.

*"under the implementation of patients self-recording pain on a lager scale."*

*should be:*

*"under the implementation of patients self-recording pain on a larger scale."*

This has been corrected (track changes line 401).

*"If a patient answer is yes, a comment for the nurses to discus the pain appears in the patient electronic medical record."*

*Should be:*

*"If a patient answer is yes, a comment for the nurses to discuss the pain appears in the patient electronic medical record."*

This has been corrected (track changes line 409).

*"...high satisfaction rate for the majority of patients stakeholders and that it provides outcome comparable to nurses pain assessment"*

*Should be:*

*"...high satisfaction rate for the majority of patients stakeholders and that it provides an outcome comparable to nurses pain assessment."*

This is corrected (track changes line 419).

*6. Patients were notified three times daily to assess their pain. At what times? Was it different per patient based on when they started or at the same time for all patients?*

All patients were notified at fixed times at 8:00 am, 14:00 pm and 22:00 pm. We adjusted the text into ‘In addition, all patients were notified three times daily fixed at 8:00 am, 14:00 pm and 22:00 pm by the application to record pain’ (track changes lines 139-141).

7. *This phrasing is confusing: "We determined the following items from the literature: design, usability, content, and workflow indexing the feedback from the patients and stakeholders"*

*I think you mean to say that you grouped feedback into these themes or categories, as has been done before (by cited papers).*

We adjusted the phrasing into ‘we grouped feedback from patients and stakeholders into the following themes determined the following items from the literature: design, usability, content, and workflow, obtained from previous research indexing the feedback from the patients and stakeholders (25-26)’ track changes lines 186-188).

8. *How come the percentages in the rows of Table 2 all add up to different amounts (below 100)? Do these represent patients who did not reply to these questions? You have the brackets wrong in the cell that's in the 10th row and 3rd column.*

Indeed, the different percentages in the rows of table 2 are due to the fact that 3 patients did not or partially complete the questionnaire. This was already mentioned in the text (track changes lines 207-210). We have now added: ‘one patient used the application, but only partially completed the questionnaire’ (track changes lines 210-211). Furthermore, we adjusted the table 2 legend with the following sentence ‘the difference in total numbers per question is explained by 3 patients who did not or partially complete the questionnaire’ (track changes line 250-251). The brackets in cell row 10, column 3 have been corrected

9. *Part of the Discussion reads: "the number of pain scores ranging from NRS ‘0 to 4’ and NRS ‘5 to 7’ recorded by patients were statistically significant higher compared with the recordings of nurses (p 0.0024, p 0.0096)"*

*This is not really written correctly. You should say "the percentage" not "the number" and the '0 to 4' range was lower while the '5 to 7' range was higher than when recorded by nurses, not both higher.*

Thank you. We have corrected the text to: ‘However, there are some differences, the percentage of pain scores ranging from ‘0 to 4’ recorded by patients with the app was lower compared with the NRS recordings of the nurses (p = 0.0024). Moreover, the percentage of pain scores ranging from ‘5 to 7’ recorded by patients with the app was higher compared with the NRS recordings of nurses (p = 0.0096) although these differences are probably not clinically relevant’ (track changes lines 334-340).

10. *Reviewer #2 has a good point, that "The results need to provide descriptive statistics-including minimum and maximum – for the number of ratings provided per patient before questionnaire completion". I do not understand your response. The reviewer is referring to statistics summarizing the number of ratings per participant using the app. By "questionnaire" in your response do you mean the app ratings or the end questionnaire? Either you are saying that the data in the app does not have time stamp (which can't be true since you provide an in-app graph of ratings over time) or perhaps you are saying the questionnaire was completed before the actual end use of the app...ie. patients used the app even after the questionnaire. This second possibility would be quite odd, but regardless you could summarize the total number of ratings done by each patient.*

We agree on this point that our answer was not clear. In this current revision we have now provided descriptive statistics for the number of ratings per patient before questionnaire completion in the Results section: ‘Indicating the experience patients have gained in using the app. The median number of pain recordings by patients before questionnaire completion was 3 with a range of 0 to 13’ (track changes lines 214-216).

11. *Later Reviewer #2 also asks "For example, were they interviewed individually or in groups?” You should put the answer to this question into your actual manuscript as it is an important point.*

We agree with the reviewer that this is important information. It was already mentioned in the manuscript. ‘Stakeholders were individually questioned during a semi-structured interview conducted by a researcher after they had the opportunity to examine the application (supporting information 3 and 4)’ (track changes lines 173-175).

12. *Unfortunately, in the regular body of the manuscript (not in tables and references) there are a number of differences between the tracked version and the cleaned version. It seems some things were adjusted in both separately. This makes it difficult to review. Some examples are on lines 170-171, 315, 352-353.*

We want apologize for making the reviewing process more complicated. We have now corrected all identified errors and carefully addressed all editorial and reviewer comments in the current track changes version of the manuscript and made an exact copy of the changes in the ‘clean version’.
